# What Builds Resilience? Sociodemographic and Social Correlates in the Population-Based LIFE-Adult-Study

**DOI:** 10.3390/ijerph19159601

**Published:** 2022-08-04

**Authors:** Elena Caroline Weitzel, Heide Glaesmer, Andreas Hinz, Samira Zeynalova, Sylvia Henger, Christoph Engel, Markus Löffler, Nigar Reyes, Kerstin Wirkner, A. Veronica Witte, Arno Villringer, Steffi G. Riedel-Heller, Margrit Löbner

**Affiliations:** 1 Institute of Social Medicine, Occupational Health and Public Health (ISAP), Medical Faculty, University of Leipzig, Philipp-Rosenthal-Str. 55, 04103 Leipzig, Germany; 2 Department of Medical Psychology and Medical Sociology, Medical Faculty, University of Leipzig, Philipp-Rosenthal-Str. 55, 04103 Leipzig, Germany; 3 Institute of Medical Informatics, Statistics and Epidemiology, University of Leipzig, Härtelstr. 16-18, 04107 Leipzig, Germany; 4 LIFE—Leipzig Research Centre for Civilization Diseases, University of Leipzig, Philipp-Rosenthal-Str. 27, 04103 Leipzig, Germany; 5 Department Neurology, Max Planck Institute for Human Cognitive and Brain Sciences, Max Planck Society, Stephanstr. 1a, 04103 Leipzig, Germany

**Keywords:** resilience, sociodemographic correlates, social network, social support

## Abstract

Resilience is closely related to mental health and well-being. Identifying risk groups with lower resilience and the variables associated with resilience informs preventive approaches. Previous research on resilience patterns in the general population is heterogeneous, and comprehensive large-scale studies are needed. The aim of our study is to examine sociodemographic and social correlates of resilience in a large population-based sample. We examined 4795 participants from the LIFE-Adult-Study. Assessments included resilience (RS-11), social support (ESSI), and social network (LSNS), as well as the sociodemographic variables age, gender, marital status, education, and occupation. The association of resilience with sociodemographic and social correlates was examined using linear regression analyses. Higher resilience was associated with female gender, married marital status, high education, and full-time occupation. Social support and social network were positively associated with resilience. Our results implicate that resilience is related to various sociodemographic variables. Social variables seem to be particularly important for resilience. We identified risk groups with lower resilience, which should be given special attention by public health policies, especially in times of crisis. Reducing loneliness and promoting social connectedness may be promising ways to build resilience in the general population.

## 1. Introduction

Dealing with adverse life events such as the COVID-19 pandemic requires resilience. According to the American Psychological Association (APA), resilience means “adapting well in the face of adversity, trauma, tragedy, threats, or significant sources of stress” [1]. The construct originates from developmental psychopathology and, analogous to the salutogenesis model, shows a shift towards resources instead of vulnerability factors [2,3]. It is closely related to well-being and mental health [4]. Thus, resilience has been shown to be protective with respect to psychological distress in the COVID-19 pandemic [5,6].

Significant characteristics enabling resilience are optimism, self-efficacy, positive emotions, and adaptive coping styles [7,8]. Some assume that resilience is a moderately stable trait [9]. However, the prevailing view is that it is rather a process that arises from an interaction of intraindividual characteristics and the environment [1,3,10,11,12]. The dependence of resilience on the sociocultural context [3] illustrates that findings from international studies or subpopulations cannot simply be transferred to the German general population. Learning more about resilience patterns and determining factors of resilience in the German general population provides implications for the prevention of mental illness and German Public Mental Health measures [4,13]. Until now, not many studies have assessed the characteristics and correlates of resilience in Germany. In our study, we aim to address this research gap by examining sociodemographic and social correlates of resilience in a large population-based sample of the German general population.

Previous research on sociodemographic correlates of resilience shows varying results. Many studies focus on at-risk populations like individuals with chronic diseases or experiencing adverse life events [14,15,16]. Only a few studies examine sociodemographic correlates of resilience at the population level showing heterogeneous results regarding gender and age [9,17,18,19]. While previous research suggests a protective effect of education on resilience [9,18], the influence of marital status and occupational situation was scarcely examined. There is a need for large-scale studies on resilience patterns in the general population, which involve a variety of sociodemographic characteristics and control for confounding influences. Our study aims to address this research gap by examining the adjusted association of resilience with important sociodemographic characteristics in a large population-based sample.

Social variables such as social support and social network are crucial for well-being and mental health [20,21,22]. Previous research identified social support as a relevant resilience resource in particularly burdened individuals [14,23,24,25]. In addition, Park et al. [26] and Wells [27] found that a larger social network was associated with higher resilience in older adults. Social variables like social support and social network are strongly interdependent and might have overlapping influences in the prediction of mental health outcomes [28]. We, therefore, aim to add to existing research by examining the independent association of social support and social network with resilience. Based on previous research, the following research questions were derived:

1. How is resilience currently distributed in the German general population?
2. Which sociodemographic characteristics are associated with resilience?
3. How are social support and social network associated with resilience?

## 2. Materials and Methods

### 2.1. Sample

Data were derived from the population-based Leipzig Research Centre for Civilization Diseases (LIFE) study, a population-based large-scale cohort study. The baseline sample consists of 10,000 Leipzig residents aged 18–79 years. Details of the LIFE-Adult-Study have been published elsewhere [29,30]. In this study, we analysed cross-sectional data of the first follow-up assessment, which included resilience for the first time and took place from 2017 to 2021 in Leipzig, Germany.

**Figure 1:**
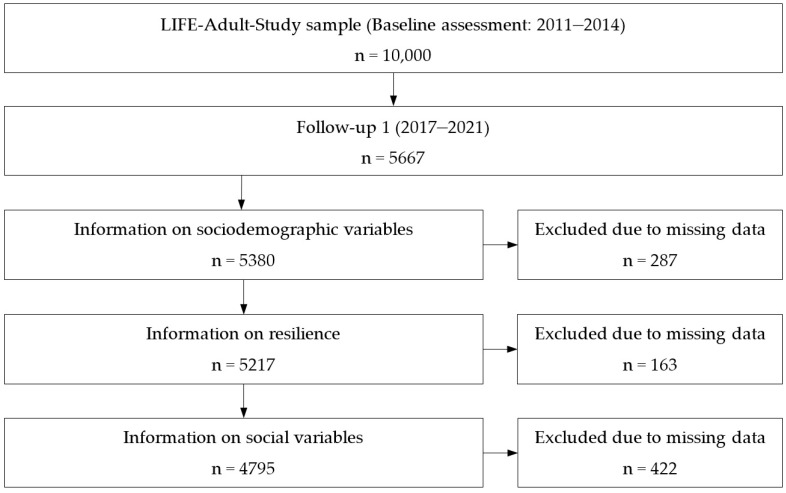
Formation of the sample.

Figure 1 shows the formation of the sample for our analyses. Follow-up data from n = 5667 participants were available. Of those, we excluded n = 287 due to missing information on relevant sociodemographic variables (age, gender, education, marital status, occupation), which resulted in a sample of n = 5380. Next, we excluded participants with missing information on resilience as measured by the RS-11 (n = 163). Further, we excluded n = 422 participants with missing information on social variables (social support and social network), which resulted in a sample of n = 4795. Missing information in resilience were less likely for individuals of female gender (*p* = 0.022), middle and high education (*p* = 0.005 and *p* < 0.001) and more likely for those married and living separately (*p* = 0.014) and retired participants (*p* = 0.023). Missing in social support and/or social network were more likely in retired participants (*p* < 0.001) and in those with other occupations (*p* < 0.001).

### 2.2. Assessments

#### 2.2.1. Sociodemographic Characteristics

Sociodemographic information included age, gender, educational and vocational qualification, marital status, and occupation. We summarized participants in three age groups (26–39 years, 40–59 years, and ≥60 years) according to Pabst et al. [31] to generate data on resilience according to age. Information on educational and vocational qualifications was classified into low, middle, and high education according to the Comparative Analysis of Social Mobility in Industrial Nations (CASMIN) educational classification [32]. Information on occupation was summarized as full-time (≥35 h), part-time (15–34 h), unemployment, retirement, and others. The limit of 35 h for full-time work corresponds to sector-specific collective agreements.

#### 2.2.2. Resilience

Resilience was assessed with the German 11-item short version (RS-11 [2]) of the Resilience Scale by Wagnild and Young [33]. The RS-11 is a unidimensional scale with exceptional psychometric parameters (Cronbach’s α = 0.91) and a valid measure of a general resilience factor [2]. The scale assesses resilience using a 7-point Likert scale from 1 = “strongly disagree” to 7 = “strongly agree”. Exemplary items are “I usually manage one way or another”, “I feel that I can handle many things at a time”, and “I can usually look at a situation in a number of ways”. All answers are added up to a sum score (range: 11–77), with higher values indicating higher resilience.

#### 2.2.3. Social Support

The German version [34] of the ENRICHD Social Support Inventory (ESSI [35]) was used to assess social support. The scale assesses aspects of perceived social support, like the availability of supportive contacts in case of problems, of people who are trusted and who provide feelings of affection and love. The ESSI consists of five items which can be answered on a 5-point Likert Scale ranging from 1 = “none of the time” to 5 = “all of the time”. Answers are summed up to a sum score ranging from 5 to 25, with higher values indicating higher social support.

#### 2.2.4. Social Network

The 6-item short version (LSNS-6) of the Lubben Social Network Scale (LSNS [36]) was used to assess the size of the social network. The scale provides quantitative information on family and friendship ties [37]. Items include the number of family members and friends, which are available for help, private talks, and with whom social contact is maintained (response options: 0 = “none”, 1 = “one”, 2 = “two”, 3 = “three or four”, 4 = “five thru eight”, and 5 = “nine or more”). A sum score is computed, which ranges from 0 to 30. Higher values indicate a larger social network.

### 2.3. Statistical Analyses

A weighting factor was computed in reference to the 2020 census data of the German general population to ensure representativeness of the study sample in terms of age and gender. The sample was weighted based on age and gender in all statistical analyses. The association of resilience with sociodemographic variables was examined with multiple linear regression analyses (model 1). The outcome variable was resilience; the independent variables were gender, age group, marital status, education, and occupation. In model 2, we extended model 1 by adding social support and social network as predictor variables to examine the influence of social variables on resilience. Regression analyses were conducted with robust standard errors and regression weights β of continuous variables were standardized for better comparability.

All analyses were performed with R [38], Rstudio [39], and the additional packages survey [40], dplyr [41], psych [42], and jtools [43]. Statistical significance was defined with *p* < 0.05.

## 3. Results

### 3.1. Descriptives

#### 3.1.1. Sample Characteristics

Sample characteristics are listed in Table 1. The mean age of the study sample was 53.62 years (*SD* = 16.66, range: 26–86 years), and 50.9% were female (*n* = 2533). Women had a mean age of 54.43 (*SD* = 16.83) and men had a mean age of 52.79 (*SD* = 16.44).

#### 3.1.2. Distribution of Resilience

Mean resilience was 60.24 (*SD* = 10.62). Table 2 shows means of resilience according to age groups and gender. Mean resilience tended to be higher among women (*M* (*SD*) = 60.71 (10.25)) than men (*M* (*SD*) = 59.67 (10.97)). Across age groups, resilience was highest in the age group 40-59 years (*M* (*SD*) = 60.51 (9.93)) for the whole sample. This trend could also be observed for men (*M* (*SD*) = 60.34 (9.88)). For women, the highest resilience was observed in the age group 26–39 years (*M* (*SD*) = 61.21 (8.99)).

### 3.2. Association of Resilience with Sociodemographic Characteristics

Results of the multiple linear regression analysis are shown in Table 3. Model 1 declared 2.7% of the variance in resilience (adjusted R^2^ = 0.027). Gender, marital status, education, and occupation were significantly associated with resilience. Female gender predicted higher resilience in reference to male gender (β^2^ = 1.648, *p* = 0.010). Single and divorced marital status were associated with lower resilience in reference to those married and living together (β^2^ = −1.941, *p* = 0.015 and β^2^ = −1.472, *p* = 0.023). High education was associated with higher resilience in reference to low education (β^2^ = 3.023, *p* = 0.024). In reference to full-time occupation, part-time occupation predicted lower resilience (β^2^ = −3.416, *p* = 0.014), as well as unemployment (β^2^ = −5.398, *p* = 0.009) and retirement (β^2^ = −2.757, *p* < 0.001). Age groups were not significantly associated with resilience.

### 3.3. Resilience and Social Variables

Social support and social network were significant predictors of resilience in model 2. Higher social support predicted higher resilience (standardized β^2^ = 2.809, *p* < 0.001) and a greater social network predicted higher resilience (standardized β^2^ = 1.758, *p* < 0.001). Model 2 declared 14.5% of the variance in resilience (adjusted R^2^ = 0.145).

## 4. Discussion

To our knowledge, this is the first study that comprehensively examined sociodemographic and social variables of resilience in a large population-based sample. The LIFE-Adult sample’s mean resilience was 60.24 (*SD* = 10.62), which is comparable to other studies [9,18].

The present study comprehensively addressed sociodemographic correlates of resilience. Previous studies on the association of resilience and gender were heterogeneous. Some studies found higher resilience in men than in women [15,18,19,44]. Others found higher resilience in women [9,23]. Our findings are in concordance with the latter and determine a significant association between female gender and higher resilience. As with Netuveli et al. [23], women also tended to have higher social support and a larger social network in our sample. When we added social determinants of resilience in model 2, the association between gender and resilience was no longer significant. The association of the female gender with higher resilience, therefore, might be explained by a better social connectedness of women in our sample. Future studies should examine underlying causes of gender differences in resilience more closely and thus contribute to an explanation of the heterogeneity of previous research.

Additionally, previous research on the association of age and resilience was heterogeneous. While some studies found an association between higher age with lower resilience [45,46], others found that resilience was higher at higher ages [9,47]. On the one hand, the onset of various chronic diseases and interpersonal losses could make resilient adaptation at an older age more difficult [45,46]. On the other hand, better emotion regulation and life experience can strengthen confidence in successfully overcoming crises and encourage proactive coping in older adults [5,47,48]. The latter was evident during the COVID-19 pandemic, with the well-being of older populations remaining relatively stable compared to the young [49]. In the LIFE-Adult sample, we observed that neither adults aged 40–59 years nor those aged 60 years and older differed significantly from those aged 26–39 years in terms of resilience. In line with Linnemann et al. [9], our results emphasize that in the general population, resilience tends to be stable across age. Nevertheless, the age of ≥60 years was associated with higher resilience in reference to those 26–39 years when social variables were added to the model. This is an interesting finding, which emphasizes the relevance of social inclusion in older age and deserves further research. Also, long-term studies are necessary that focus on individual trajectories of resilience over the lifespan.

In our study, marital status was associated with resilience. We found that single and divorced participants reported lower resilience than those who were married and living with their spouses. These findings are in line with Beutel et al. [50] and Beutel et al. [51], who found higher resilience in those living in a partnership. An explanation might be the provision of social support by the spouse. This hypothesis is supported by the fact that marital status was no longer a significant predictor of resilience when we included social support and social network in model 2. Accordingly, interventions to support the social inclusion of single and divorced persons should be provided.

We further found that widowed participants had similar resilience to married participants who were living together. This contrasts with Kocalevent et al. [18], who find lower resilience in widowed participants, and might be explained by a different methodological approach. In contrast to Kocalevent et al. [18], we controlled for confounding factors like education, occupation, and gender. Our results provide a significant extension of previous research: although spousal loss negatively affects mental health [22], it does not seem to be accompanied by lower resilience. The role of resilience in coping with widowhood and the impact on mental health should be explored in future studies.

Previous research suggests a protective effect of education with regard to resilience. Although Laird et al. did not find any association of resilience with education [52], most studies found higher resilience in those with higher education [9,17,18,53,54]. In concordance with previous research, we found that high education was associated with higher resilience compared to low education. Interestingly, the association between education and resilience did not remain significant when adding social support and social network to the analysis. Further studies should take a closer look at resilience patterns regarding education and whether social integration is a relevant modulating factor.

Until now, only a few studies examined the association of occupation with resilience. Kocalevent et al. [18] and Kunzler et al. [17] found that employment was associated with higher resilience than unemployment. In other studies, previous unemployment was associated with lower resilience [50,51]. We took a closer look at the association between current occupation and resilience in the German general population. In line with Kunzler et al. [17], we found that part-time working and unemployed participants had lower resilience compared to full-time working participants. To our knowledge, we were the first to examine resilience in retired compared to full-time working individuals. We have observed for the first time that retired individuals were likely to show lower resilience. These results extend previous research and emphasize the relevance of professional life for resilience. One possible explanation is the higher income and higher social status of full-time work, which has been associated with resilience in other studies [18,55]. Our results implicate that special attention should be given to the needs of those without satisfactory employment or upon retirement.

In addition, we examined the association of social support and social network with resilience. Previous research found that both variables were associated with resilience [14,23,25,26,27]. Until now, it has been unclear how these variables are associated with resilience when controlled for each other. The present study shows that both variables were independently associated with resilience in the German general population and that the explained variance in resilience increased substantially when social variables were added to the model. Social support can broaden the range of adaptive coping strategies when facing adversity and support emotion regulation [56]. Our results demonstrate that social inclusion is highly relevant for resilience and that both quantitative and qualitative aspects of interpersonal relationships are important. This is an important finding because this has implications for building resilience in the general population and German Public Mental Health measures. For example, in terms of prevention, interpersonal networking can be expanded at the local level through community activities and meeting places, like neighborhood cafés. At the same time, strengthening social relationships could help promote resilience among at-risk groups. For example, social participation could be targeted more strongly for people with existing mental illnesses. These report lower social inclusion than healthy individuals [57] and could particularly benefit from strengthening social resources and increasing resilience. Particular attention should also be paid to people who are likely to experience changes in their social environment, e.g., due to job loss, retirement, or divorce. For example, a reduction in the size of the social network upon retirement could be counteracted by a greater promotion of volunteerism.

Together, the sociodemographic variables explained a limited amount of variance in resilience. The inclusion of social variables has led to an increase in explained variance in resilience, particularly regarding stress. This indicates that other factors influencing resilience, such as environmental factors, should be examined. Since resilience describes good adaptation to adversity, it is likely that exposure to adversity might have a relevant impact. Thus, loss of experience or a severe chronic illness could impair resilience or, conversely, successfully handling a crisis could strengthen one’s resilience [3]. Other studies suggest that psychological factors such as self-efficacy might manifest in greater resilience [58]. The association of further intrapsychic and external factors with resilience should be addressed in future studies.

### Strengths and Limitations

To our knowledge, this is the first study to give comprehensive insight into sociodemographic and social correlates of resilience based on a large population-based sample. Still, our analysis is not without limitations. Data on resilience were only available for the follow-up assessment of the LIFE-Adult-Study. The results are therefore limited to a cross-sectional caption on resilience patterns in the German general population. Further longitudinal studies should examine resilience trajectories over time, particularly regarding the association of resilience with age. It also cannot be ruled out that contact restrictions due to the pandemic might have affected information on social support and social network. Further, the present study does not allow any generally valid categorical statements to be made about high or low resilience. Future studies should investigate the extent to which resilience can be classified as low or high resilience.

## 5. Conclusions

Resilience is relevant for mental health and well-being. Identifying correlates of resilience informs preventive approaches. We found that gender, marital status, education, and occupation are related to resilience. Our findings further illustrate that interpersonal relationships are crucial for resilience and provide starting points for interventions. In times of crisis, social inclusion of at-risk groups should be given special attention to facilitate resilient adaptation. Community-based services, in particular, could be effective here by expanding low-threshold activities and offering meeting places.

## Figures and Tables

**Table 1:** Sample characteristics.

Variable	Total *n* = 4795		Women *n* = 2541		Men *n* = 2254	
***Sociodemographic variables***						
Age, *M* (*SD*)	53.62	(16.66)	54.43	(16.83)	52.79	(16.44)
Age group, *n* (%)						
26–39 years	162	(25.35)	80	(24.19)	82	(26.55)
40–59 years	1704	(37.54)	947	(36.72)	757	(38.39)
≥60 years	2929	(37.11)	1514	(39.09)	1415	(35.06)
Marital status, *n* (%)						
Married (living together)	3066	(53.06)	1470	(50.50)	1596	(55.72)
Married (living seperatly)	95	(1.96)	54	(1.95)	41	(1.98)
Single	650	(30.00)	331	(27.25)	319	(32.85)
Divorced	567	(9.05)	358	(10.78)	209	(7.25)
Widowed	417	(5.92)	328	(9.51)	89	(2.19)
Education, *n* (%)						
Low	277	(4.91)	165	(6.86)	112	(2.89)
Middle	2637	(57.36)	1512	(57.18)	1125	(57.55)
High	1881	(37.73)	864	(35.95)	1017	(39.57)
Occupation, *n* (%)						
Full-time (≥35 h)	1746	(51.35)	825	(42.29)	921	(60.74)
Part-time (15–34 h)	389	(11.71)	326	(17.67)	63	(5.52)
Unemployed	88	(2.65)	43	(2.68)	45	(2.62)
Retired	2323	(27.87)	1193	(30.19)	1130	(25.47)
Other	249	(6.42)	154	(7.16)	95	(5.65)
***Social variables***						
Social support, *M* (*SD*)	22.38	(3.45)	22.53	(3.36)	22.22	(3.53)
Social network, *M* (*SD*)	17.91	(5.39)	18.12	(5.21)	17.70	(5.56)

*Notes*. % are weighted by age and gender according to census data, *n* are unweighted count. Social support and social network were measured with the ENRICHD Social Support Inventory (ESSI) and the Lubben Social Network Scale (LSNS).

**Table 2:** Mean resilience by age group and gender.

	Total *n* = 4795		Women *n* = 2541		Men *n* = 2254	
	*M*	*SD*	*M*	*SD*	*M*	*SD*
Total	60.24	10.62	60.71	10.25	59.67	10.97
Age group						
26–39 years	59.84	10.69	61.21	8.99	58.57	12.00
40–59 years	60.51	9.93	60.69	9.98	60.34	9.88
≥60 years	60.12	11.25	60.43	11.22	59.76	11.28

*Notes.* Descriptive results are weighted by age and gender according to census data. Resilience was measured with the RS-11 (range: 11–77).

**Table 3:** Results of the multiple linear regression analyses ^1^.

Predictor Variable	Model 1				Model 2			
	β	95% CI Lower Bound	95% CI Upper Bound	*p*-Value	β	95% CI Lower Bound	95% CI Upper Bound	*p*-Value
Intercept	59.904	56.718	63.090		58.446	55.483	61.409	
***Sociodemographic variables***								
Age group, ref. 26–39 years								
40–59 years	−0.276	−2.192	1.639	0.777	1.314	−0.481	3.109	0.151
≥60 years	0.555	−1.579	2.689	0.610	2.843	0.830	4.856	**0.006**
Gender, ref. male								
Female	1.648	0.390	2.906	**0.010**	1.060	−0.080	2.200	0.068
Marital status, ref. married and living together								
Married, living seperatly	−0.724	−3.066	1.617	0.544	0.738	−1.544	3.020	0.526
Single	−1.941	−3.499	−0.382	**0.015**	0.144	−1.397	1.686	0.854
Divorced	−1.472	−2.736	−0.208	**0.023**	0.781	−0.395	1.956	0.193
Widowed	0.049	−1.605	1.704	0.963	1.003	−0.482	2.489	0.186
Education, ref. low								
Middle	2.087	−0.515	4.690	0.116	1.122	−1.238	3.483	0.351
High	3.023	0.401	5.644	**0.024**	1.598	−0.767	3.964	0.184
Occupation, ref. full-time (≥35 h)								
Part-time (15–34 h)	−3.416	−6.142	−0.689	**0.014**	−3.145	−5.627	−0.663	**0.013**
Unemployed	−5.398	−9.430	−1.366	**0.009**	−3.318	−6.429	−0.208	**0.037**
Retired	−2.757	−4.149	−1.364	**<0.001**	−2.311	−3.620	−1.001	**0.001**
Other	−1.674	−4.981	1.633	0.321	−2.028	−5.287	1.231	0.223
***Social variables***								
Social support					2.803	2.270	3.336	**<0.001**
Social network					1.758	1.257	2.258	**<0.001**

*Notes*. Results are weighted by age and gender according to census data. Resilience, social support, and social network were measured with the RS-11, the ENRICHD Social Support Inventory (ESSI), and the Lubben Social Network Scale (LSNS). β of continuous predictors (social support, social network) are standardized; β of categorial predictors describe estimated mean difference of resilience to reference group. Bold *p*-values indicate significance. ^1^ Multiple linear regression analysis was performed with resilience as outcome and sociodemographic variables as predictor variables. In model 2, social variables were added as predictor variables.

## Data Availability

The dataset analysed during the current study is available from the corresponding author upon reasonable request.
